# Independent Dental Journalism and Its Use

**Published:** 1891-03

**Authors:** 


### INDEPENDENT DENTAL JOURNALISM AND ITS USE.

The employment of the term *independent* as applied to dental journalism has excited opposition and criticism from the moment of its adoption. Why is this? Why is it that a term which when used in connection with general journalism means freedom of expression of thought should excite when it comes to be applied to an organ of dental opinion a feeling of antagonism?

The argument is used that because a professedly independent organ requires for its existence an income derived from subscribers and advertisers, and is also liable to suffer in these resources by the adverse opinion of its readers, that, therefore, it can have no claim to independence. If that were the restriction of the value of the word the criticism would have force.

In this sense it is true that every man who employs his muscle or his brain to secure the means to support his physical life is dependent. But he may be deprived for a time of all that makes life agreeable and yet maintain throughout that independence of mind which constitutes true manliness. He will be free to think what is true and do what is best, and in this intelligent age the result of this kind of independence must come out victorious.

It is this manliness applied to the needs of the dental profession that is required, and it is this quality which independent journalism claims to hold.

While dependent for sustenance upon its patrons, like an individual man, it stands on higher ground. It views our profession, or our specialty, as many call it, with distinctiveness of character and of organization. It finds a body of at least twenty thousand active intelligent members without a current literature of its own to furnish opportunity for the full expression of such views as are favorable to the growth of the higher sentiments which should be under development in so large a body of men.

So forcible and general is this sentiment that no distinct class will rest long without some form of leadership of opinion and without some medium that will incline to raise the general mass above the influence of the meaner instincts which are connected with the pursuit of wordly existence. After supplying the current knowledge of science, of facts, and of procedures relating to our special work, independent journals should have as their chief end the stimulation of those impulses which tend to develop professional spirit. However faint the expression may be, however slight the influence at first may appear, the movement of arousing the sentiment of solidarity will go on, and the undermining influences which it is too plain have been leading to the decay of true professional life will die out.

The higher effort of raising the aims of the dental worker above those things pertaining to the lower elements of his calling to those which concern his mental growth and the cultivated breadth of which his specialty is capable belongs to that form of dental literature which will grow up through the care of journals published under the control of practising dentists.

It is for these reasons that this journal, which is the outcome of the deeply-seated sentiment that dentistry needed to have its literature under its own control, espouses the cause of the kind of independent journalism above delineated, and calls upon dentists everywhere to support any effort to foster a periodical literature which is liberal in tone and independent in spirit. ***

